# Environmental spatial heterogeneity of the impacts of COVID-19 on the top-20 metropolitan cities of Asia-Pacific

**DOI:** 10.1038/s41598-021-99546-9

**Published:** 2021-10-13

**Authors:** Ghaffar Ali, Sawaid Abbas, Faisal Mueen Qamer, Syed Muhammad Irteza

**Affiliations:** 1 College of Management, Shenzhen University, Shenzhen, 518060 Guangdong China; 2 Department of Land Surveying and Geo-Informatics, The Hong Kong Polytechnic University, Kowloon, Hong Kong; 3 International Center for Integrated Mountain Development (ICIMOD), Kathmandu, 44700 Nepal; 4 Remote Sensing, GIS and Climatic Research Lab (RSGCRL), National Center of GIS and Space Applications, University of the Punjab, Lahore, Pakistan

**Keywords:** Environmental sciences, Environmental impact

## Abstract

This study investigated the environmental spatial heterogeneity of novel coronavirus (COVID-19) and spatial and temporal changes among the top-20 metropolitan cities of the Asia-Pacific. Remote sensing-based assessment is performed to analyze before and during the lockdown amid COVID-19 lockdown in the cities. Air pollution and mobility data of each city (Bangkok, Beijing, Busan, Dhaka, Delhi, Ho Chi Minh, Hong Kong, Karachi, Mumbai, Seoul, Shanghai, Singapore, Tokyo, Wuhan, and few others) have been collected and analyzed for 2019 and 2020. Results indicated that almost every city was impacted positively regarding environmental emissions and visible reduction were found in Aerosol Optical Depth (AOD), sulfur dioxide (SO_2_), carbon monoxide (CO), and nitrogen dioxide (NO_2_) concentrations before and during lockdown periods of 2020 as compared to those of 2019. The highest NO_2_ emission reduction (~ 50%) was recorded in Wuhan city during the lockdown of 2020. AOD was highest in Beijing and lowest in Colombo (< 10%). Overall, 90% movement was reduced till mid-April, 2020. A 98% reduction in mobility was recorded in Delhi, Seoul, and Wuhan. This analysis suggests that smart mobility and partial shutdown policies could be developed to reduce environmental pollutions in the region. Wuhan city is one of the benchmarks and can be replicated for the rest of the Asian cities wherever applicable.

## Introduction

Novel coronavirus 2019 commonly known as COVID-19 has become the additive factor of everyday life across the globe. A lot of reporting, analysis, and suggestions are being available and published recently^1–3^. According to the latest statistics by the World Health Organization^4^, over 200 million people have been affected and the death toll rises to almost 4.6 million by September 2021^4^. And this trend of the outbreak from epidemic to pandemic is continued and all efforts have been mobilized to flatten the curve. This led to various lockdown implementations in different countries and cities (almost 216), starting from Wuhan, China on January 21, 2020^5^. Since then, lockdown is being implemented in most of the Asia-Pacific countries such as partial lockdown in Bangladesh, China, India, Japan, Pakistan, South Korea, and Thailand. Asia-Pacific region includes most of the countries in East, West, South Asia, and Oceania with proximity to Western Pacific Ocean^6^. Besides its negative impacts on daily life in terms of social, economic, health, environmental, and development forms, few co-benefits on our natural environment may also be observed. It is hard to grasp the notion of co-benefits of COVID-19; however, changes are actual and evident.

Having said that, some studies have reported the air quality correlated with COVID-19^7–10^, some investigated its correlation with weather^11,12^; likewise, an interesting study compiled the information released by National Aeronautics and Space Administration (NASA) on pollution across the globe^13^ based on the maps released by the NASA and European Space Agency (ESA). Another study analyzed the situation in Wuhan and other 122 cities of China^5^, and global bird eye-view^14^. Nonetheless, some original analysis of temporal change in the natural environment with a comparative breakdown of the pollutants is still missing, especially, in many highly polluted metropolitan cities of the Asia-Pacific. As we know that, 99 out of the 100 world’s most polluted cities are present in the Asian region^15^, therefore, it is necessary to understand the environmental dynamics of such metropolitan cities relating to COVID-19. Moreover, this wave of lockdown following pandemic is interlinked with the mobility feature. Restriction on mobility resulted in unprecedented reductions in deadly air pollution around the world^16^. Quantifying the changes in air pollution during the lockdown period may provide a unique opportunity to understand the types, patterns, and origins of air pollution by comparing pre-lockdown with lockdown and/or post-lockdown conditions^17^. Keeping in view all these facts and research gap, this study investigated the environmental spatial heterogeneity of COVID-19 and spatial and/or temporal changes among the top-20 (now onwards T20) metropolitan cities of the Asia-Pacific.

To the best of our knowledge, no such study has been conducted thus far that analyses the environmental changes and spatial heterogeneity of COVID-19 in the T20metropolitan cities of the Asia-Pacific. Moreover, this study explains the comparative assessment of important yet selected greenhouse gases (GHGs) emissions before and during lockdown amidst COVID-19. The novelty of the research lies in analyzing the big data, linking environmental changes with COVID-19, and connecting mobility data before and during the lockdown. Therefore, the results reported here are novel, timely, and may help policymakers to understand the lockdown policies accordingly to understand co-benefits and heterogeneity of COVID-19.

## Results

**Figure 1:**
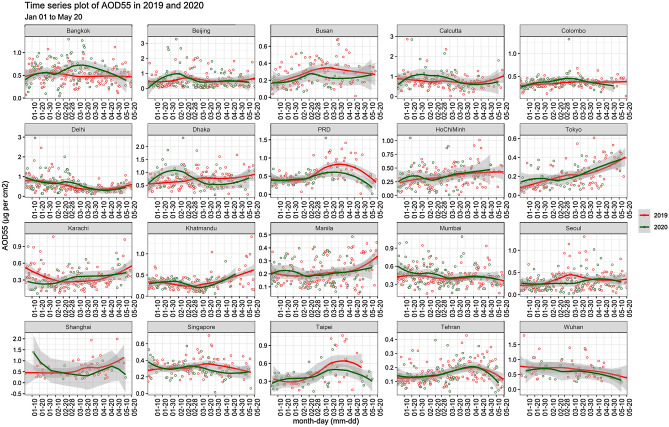
Comparison of daily changes in AOD concentration of the cities in 2019 and 2020.

Metropolitan status is achieved by any city if its agglomeration index and core population exceed 50,000 or above^18^. The first COVID-19 lockdown in the world was imposed in Wuhan city on 21st January 2020 and the latest was in Singapore, on 7th April 2020^19^. Keeping that in view and spatial heterogeneity, we have selected the metropolitan cities of the Asia-Pacific especially from middle- and low-income countries. These cities are listed as top metropolitan cities^16^ and most of them are among top in the list of most polluted cities. “Spatial heterogeneity is defined either as the variation in space in distribution of a point pattern, or variation of a qualitative or quantitative value of a surface pattern”^20^. Table 1 shows the socio-economic characteristics of the selected cities. The analysis showed that the highest levels of AOD were found in Calcutta, India (2.86) on 14th January 2019 whereas the lowest levels of AOD  were observed in Ho Chi Minh, Vietnam (0.013) on 21st March 2019. Likewise, the highest levels of AOD were found in Beijing, China (26% change) on 7th February 2020 while the lowest levels of AOD were recorded in Ho Chi Minh (0.01% change) on 21st March 2020. According to the analysis, top four cities for AOD in 2019 were Calcutta, Beijing, Busan, and Tokyo, however, the situation was slightly changed in 2020 during the lockdown period. During lockdown highest levels of AOD were found in Beijing, Calcutta, Delhi, and Dhaka (Fig. 1).

**Table 1:** Demographic characteristics of the selected cities^21^.

City	Total area (Sq.km)	Population (million)	Urbanization rate (%)	Growth rate (%)
Bangkok	1568	10.7	1.73	1.74
Beijing	16,410	20.8	2.4	2.12
Busan	766	3.4	0.3	0.02
Calcutta	205	14.9	2.2	0.84
Colombo	37.2	0.6	0.85	1.06
Delhi	1484	31.1	2.94	2.94
Dhaka	1500	21.7	3.17	3.50
Hong Kong in the Pearl River Delta	1050	7.5	0.82	0.84
Ho Chi Minh	2061	8.8	2.98	2.73
Tokyo	2187	37.3	− 0.13	− 0.14
Karachi	3778	16.2	2.53	2.27
Kathmandu	564	1.47	3.15	3.4
Manila	619	14.1	1.98	1.69
Mumbai	603	20.6	2.37	1.26
Seoul	605	9.9	0.32	0.04
Shanghai	6340	27.7	2.42	2.72
Singapore	700	5.9	1.39	0.84
Taipei	271	2.7	0.8	2.3
Tehran	1700	9.2	1.71	1.36
Wuhan	1528	8.4	2.39	1.3

The lowest levels were observed in Ho Chi Minh, Delhi, Taipei, and Wuhan cities in 2019. On the contrary, Ho Chi Minh, Tehran, Bangkok, and Shanghai were the top among lowest level cities respectively, from January to May 2020. Busan, Calcutta, and Tokyo cities were positively impacted during the lockdown and reaped more environmental benefits than other metropolitan cities (Fig. 1).

**Figure 2:**
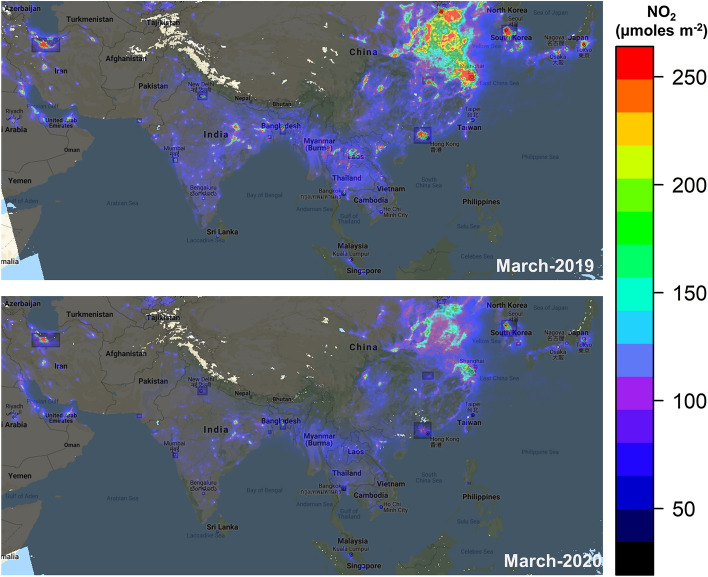
Spatial patterns indicating a drastic drop in average NO_2_ concentration in March 2019 and 2020 in the Asia-Pacific region. Maps were created through Google Earth Engine (https://earthengine.google.com/V.2020) by a co-author, Sawaid Abbas.

**Figure 3:**
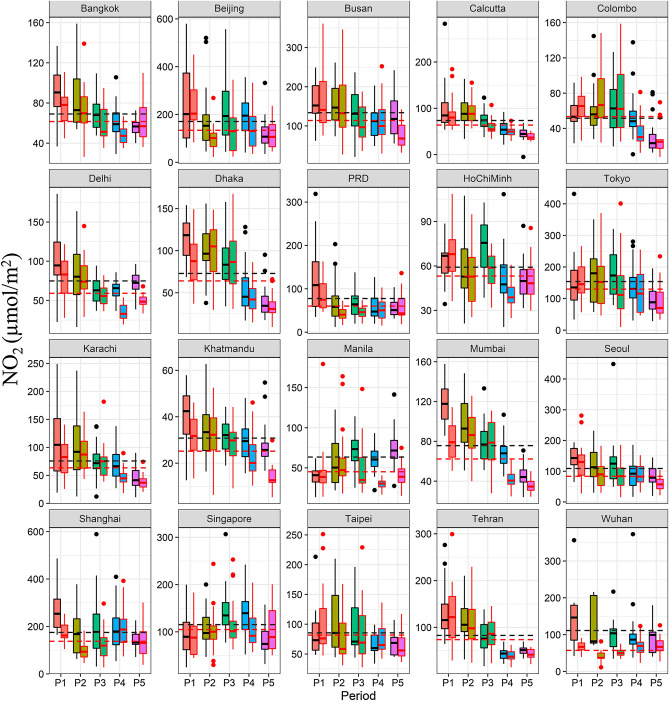
Periodic changes of NO_2_ concentration of the cities in 2019 and 2020 during the P1 (Jan 1–Jan 22), P2 (Jan 23–Feb 22), P3 (Feb 23–Mar 22), P4 (Mar 23–Apr 22) and P5 (Apr 23–May 22). The black and red dotted lines indicate mean NO_2_ concentration (Jan 1–May 22) in 2019 and 2020 and the dots indicate outliers, respectively.

Results of NO_2_ interestingly showed a mix of environmental spatial heterogeneity. A maximum level of NO_2_ was observed in Shanghai, China which was (588.953 µmol/m^2^), on 17th March 2019 whereas the minimum level of NO_2_ was recorded in Calcutta (5.06 µmol/m^2^), on 2nd May 2019. Moreover, the maximum level of NO_2_ was observed in Beijing city (20%), on 17th January 2020, as compared to 2019, which then decreased to almost 95% during the lockdown period. The minimum level in Kathmandu, Nepal showed a change of almost 85%, on 9th May 2020 as compared to the 2019. The topmost NO_2_ emitting cities in 2019 were Beijing, Wuhan, Shanghai, and Delhi; whereas during the lockdown of 2020 top cities were Beijing, Shanghai, Tokyo, and Seoul (Fig. 2). The lowest amount of NO_2_ was recorded in Calcutta, Colombo, Kathmandu, and Karachi in 2019; whereas in 2020 these three cities (Kathmandu, Colombo and Tehran) made to the list of lowest levels. Almost 50% NO_2_ emission reduction was observed in Wuhan city (Fig. 3).

**Figure 4:**
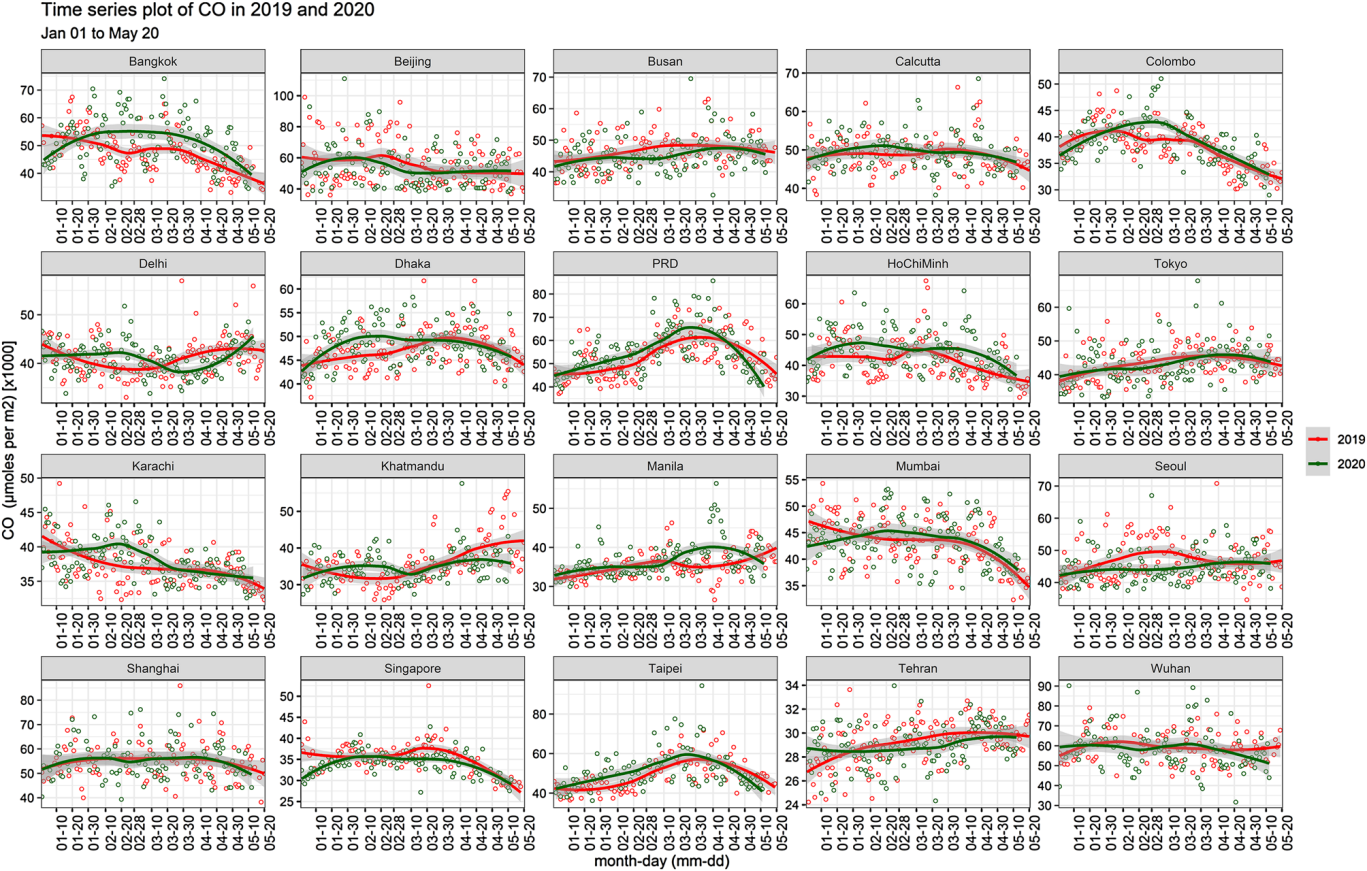
Comparison of daily changes in CO concentration of the cities in 2019 and 2020.

Considering another important air pollutant CO, Beijing was the highest city with 98.97 × 10^3^ µmol/m^2^ CO emissions on 3rd January 2019 and the lowest was in Tehran city of Iran with CO emissions (24.21 × 10^3^ µmol/m^2^) recorded on 2nd January 2019. Given the results of this analysis, CO emissions were rather increased in Beijing (10%) as per observations made on 28th January 2020 which decreased to almost 65% during March, 2020 (lockdown period). Furthermore, the lowest CO emissions remained in Tehran with (21% change) on 22nd March 2020 as compared to the previous year. The top most CO emitting city in 2019 was Beijing followed by Wuhan, Shanghai, and Delhi, and during the lockdown of 2020 Beijing was followed by Wuhan, Shanghai, and Calcutta (Fig. 4). The lowest levels of CO in 2019 were from Tehran, Kathmandu, Bangkok, and Colombo, and during the lockdown of 2020 were from Tehran, Colombo, Karachi, and Wuhan.

Figure S1 showed the results of SO_2_ emissions from the T20 cities. The maximum level of SO_2_ was observed in Kathmandu (1362.43 × 10^3^ µmol/m^2^) on 12th April 2019, and the minimum level was observed in Beijing (124.12 × 10^3^ µmol/m^2^) on 23rd January 2019. Similarly, the maximum level of SO_2_ was recorded in Dhaka and Bangladesh (with a 42% increase) on 22nd April 2020, and the minimum level of SO_2_ in Seoul which was (87% decrease), on 17th January 2020. During 2019, the highest levels of SO_2_ were in Kathmandu, followed by Calcutta, Dhaka, and Singapore, and during the lockdown of 2020 was Dhaka (42%), followed by Calcutta (23%), Hong Kong (21%), and Beijing(19%) cities (Fig. S1). SO_2_ was lowest in Beijing, Delhi, Wuhan, and Bangkok during 2019 which was a normal year than 2020. Seoul, Beijing, Busan, and Shanghai were among the top four cities that emitted the lowest SO_2_ emission during the lockdown of 2020.

**Figure 5:**
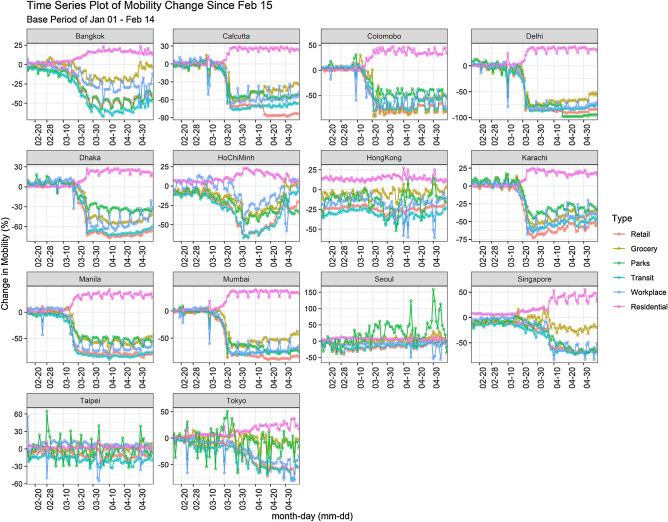
Patterns of changes in mobility in different cities due to lockdown implementation. The anomalies are computed with respect to base period from 1st January to 14th February 2020.

Air pollution is robustly interconnected with the mobility of people and other modes of transportation^22^. We considered six modes of mobility, i.e., transit, parks, residential, retail, grocery, and workplace (Fig. 5). According to Google mobility data^23^, mobility in the urban center in Pakistan went down by 75% as a result of nation-wide lockdown. The minimum mobility (-38%) in Seoul was observed on 25th February 2020, however, mobility in the parks reached its maximum (158%) on 30th April 2020. While minimum mobility in parks was noted in Delhi on 16th April 2020 (-98%). In the residential sector, maximum mobility was recorded in Singapore on 1st May 2020 (55%) and minimum mobility in the residential sector was documented in Calcutta on 11th March 2020 (-3%). In the transit sector, maximum mobility was observed in Colombo on 10th March 2020 (12%). And minimum mobility was recorded in Manila on 10th April, 2020 (-90%). According to Google mobility data, 90% movement was reduced till mid-April, 2020 in the Asia-Pacific region and it is worth to note that most of the populated cities of the world are in Asia-Pacific.

## Discussion

Main purpose of this study was to investigate the spatio-temporal heterogeneity of environmental changes due to COVID-19 lockdowns among the top-20 selected metropolitan cities of the Asia-Pacific. Different cities experienced lockdown phases from time to time^24^, we, however, used a time bracket and studied that time frame to observe and analyze the changes compared to the reference year (2019). Specifically, the changes in the air quality during the lockdown period can be classified into three types of changes (less than 10%, less than 25%, and more than 40%). Firstly, cities such as Colombo, Dhaka, Singapore, Taipei, and Tehran showed less than 10% change in NO_2_ emissions (mean annual value) during the lockdown compared to the reference year, 2019. Secondly, Bangkok, Busan, Delhi, Kathmandu, Karachi, Mumbai, PRD Hong Kong, Seoul, Shanghai, and Tokyo showed changes between 10 and 25% during the studied period. Only Beijing, Manila, and Wuhan were the cities that portrayed significant changes in the air quality as compared to the reference year. Wuhan remained at the top among all the metropolitan cities studied. Wuhan was under complete lockdown unlike Seoul and Shanghai, which is the reason why it reaped plenty of environmental benefits in the form of air pollution reduction. Our results are also similar to those studied by^25^ in different cities of China. Another study was conducted at the global level and found the same results of CO reduction^26,27^.

Our study highlighted an interesting finding that small metropolitan cities such as Colombo and Taipei were not significantly influenced by the lockdown considering air quality changes as compared to the large metropolitan cities (Beijing, Manila, Seoul, and Wuhan), and this result is contradictory to a study^28^ carried out to investigate air quality impacts during the lockdown in the Middle East metropolitan cities. Dhaka is one of the most densely populated and economically vibrant metropolitan cities; however, it did not show significant changes as per our analysis, and the results are in line with this study conducted in Bangladesh^29^. This study also found that in Bangladesh, lockdown is linked to the reduction in air pollution specifically, in Dhaka city of Bangladesh, but insignificantly^30^. Comparative analytical results showed that Wuhan and Shanghai cities especially made significant improvements in terms of AOD, NO_2_, and CO emission reductions during the lockdown^31^. Wuhan made it to the lowest emissions list of 2020 from the top as in 2019. Beijing and Wuhan again showed a variant environmental spatial heterogeneity during the lockdown period which indicate the positive impacts of COVID-19 lockdown. Moreover, it helps to understand the environmental conglomeration in different cities of Asia. It is important to note that the changes in concentrations of the pollutants and their response to lockdown implementation may vary as a function of characteristics of the city including population, urbanization or industrialization, growth rate as well as local meteorological conditions.

## Conclusions

The purpose of this study was to highlight that COVID-19 had many adverse impacts on different sectors of the economies, but environmental benefits were also reaped in the form of air pollution reduction knowingly or unknowingly. Reduction in the pollutant from east to west with time since the outbreak indicates a clear pattern and lack of timely actions. Moreover, response actions of different governments and response of residents portray sequential patterns of priorities. It is interesting to conclude that almost 50% NO_2_ emission reduction is observed in Wuhan in 2020 compared to 2019. Moreover, Beijing, Wuhan, Calcutta, and Seoul showed a significant reduction in emissions levels during the lockdown period. Dhaka, Tehran, and Colombo remained in the lowest levels of change regarding overall air pollution before and during the lockdown. The rest of the metropolitan cities remained mid-level emission emitters. Therefore, a visible pattern of environmental spatial heterogeneity was found in the Asian metropolitan cities before and during the lockdown. However, the COVID-19 lockdown made it further visible than before. This portrays that reducing few activities in the largest metropolitan cities of Asia may help to gain sufficient environmental benefits, and then some action plans should be crafted. COVID-19 lockdown economically proved to be negative but environmentally positive for almost all of the studied cities. This co-benefit analysis suggests that smart mobility and temporal shutdown policies could be developed to reduce environmental pollutions. Wuhan city is one of the benchmarks and can be replicated for the other Asian cities wherever applicable. Further studies may include/exclude other cities of Asia-Pacific to study the changes. Such impacts using new methods can also be tested to observe the changes.

## Methodology

### Study sites

The impact of lockdown varied from east to west with different lockdown periods and relevant policies in the Asia-Pacific region. Apart from a wall-to-wall assessment of air quality of the whole region, we also analyzed the patterns of changes in 20 metropolitan cities of the region including Bangkok, Beijing, Busan, Calcutta, Columbo, Delhi, Dhaka, Ho Chi Minh, Karachi, Kathmandu, Manila, Mumbai, Pearl River Delta, Seoul, Shanghai, Singapore, Taipei, Tehran, Tokyo, and Wuhan. The city-specific assessment aimed to reveal the geographic spread of the pandemic and response of governments regarding lockdown initiatives. Earth Observation-based monitoring systems are being very efficiently used to understand the impact of the COVID-19 spread on the environment in the short- and long-runs^32–34^. The study area map indicating locations and geographic coverage of the study sites can be consulted in Fig. S2 (Supplementary file).

### Data processing and analysis

We analyzed carbon monoxide (CO), nitrogen dioxide (NO_2_), sulphur dioxide (SO_2_), and Aerosol Optical Depth at 550 nm (AOD), and mobility before and during COVID-19 lockdown. The environmental datasets were processed and obtained using the cloud computing platform, Google Earth Engine, mobility data was acquired from Google, and the analysis was performed in R.

The NO_2_, SO_2_, and CO data products were derived from the TROPOspheric Monitoring Instrument (TROPOMI) onboard Sentinel 5P. Sentinel 5P enables a new era of satellite-based mapping and monitoring of atmospheric pollutants and gasses at global and regional scale. We used the level 3 products, available in the Google Earth Engine, which were subsequently masked for good quality pixel by using the relevant quality control tags of the data (S5P-GEE, 2020). These datasets were converted into measurement units of µmol/m^2^ before exporting the results at ~ 1 km spatial resolution. The AOD measurements used in this study were derived from the MODIS product (MCD19A2-V6)^35^ which is a combined product of MODIS sensors onboard Terra and Aqua satellite. It is based on the MAIAC (Multi-angle Implementation of Atmospheric Correction) algorithm and provide daily global coverage of AOD at 1 km spatial resolution. The data was screened for good quality pixels and the pixel tagged with poor quality indicators were masked out. It is important to note that the products have been spatially averaged over the hot spot region of the corresponding cities for time series assessment. Therefore, an underlying limitation of the datasets and the analysis could be the coarse spatial resolution of the imagery and spatial averaging which must be considered while assessing the pollutant concentration at local scale.

We used the monthly mean image of NO_2_ in 2019 to demarcate the hotspot areas by drawing a confounding rectangle around the high concertation pixels (Fig. S1). The environmental data analysis consists of two components—i) time series analysis from January 01 to May 22 for the year 2019 and 2021, and ii) periodic changes in the five different periods and their significance difference with average values during January 2019. The statistical significance between the periods was tested using the non-parametric Wilcoxon Rank test. The temporal patterns were also fitted using a Local Polynomial Regression Fitting (loess) algorithm to show the overall changes over time. Considering the first outbreak of the pandemic and implementation of lockdown strategies among the cities, the analysis timeframe was divided into five consecutive periods—P1 (January 01 to January 22), P2 (January 23 to February 22), P3 (February 23 to March 22), P4 (March 23 to April 22) and P5 (April 23 to May 22). We adopted several frameworks of assessments at daily, weekly, monthly and the periodic intervals. For comparative assessment the weekly summaries could have been messier and more difficult to explain while the monthly summaries may not clearly present the lockdown scenarios and normal period before the pandemic effect. For instance, a lockdown in Wuhan during the end of the January might result in a drastic drop of pollutants which can significantly influence the monthly averages and obscure the true picture. Therefore, the five time periods were meticulously selected by considering the major lockdown implementations in the region. However, it may still not be appropriate for some cities, therefore, a daily time series along with summaries of pollutants levels for the first five month of 2019 and 2020 have been analyzed.

The loss or gain (equation is given below) in the environmental pollutants were calculated by normalizing the difference between the control period (P1) and the other post-pandemic or periods associated with lockdown implementation or relaxations (P2, P3, P4, and P5). Positive values show normal or high concentration conditions while negative values indicate a decline in the pollutants.

$$ Loss\;or\;Gain{ }\left( {\text{\%}} \right) = { }\frac{{\sum Pollutants_{p} { } - { }\sum Pollutants_{c} }}{{\sum Pollutants_{c} }}{ } \times 100 $$

$Pollutants_{p}$ and $Pollutants_{c}$ represents the concentration of the pollutants during and/or post-pandemic period and during the control or normal period, respectively.

The google community mobility data calculated the changes in mobility patterns with reference to the baseline median value for each weekday obtained during the five weeks period (January 03 to February 05, 2020). All the processed and raw datasets can be obtained from the supplementary materials.

## Supplementary Information

Supplementary Information 1.

Supplementary Information 2.

## Data Availability

The environmental datasets were processed and obtained using the cloud computing platform, Google Earth Engine (available online (https://earthexplorer.usgs.gov/), mobility data was acquired from Google. All other data sets are available in the supplementary folder.
