# Supplementary material for: Environmental spatial heterogeneity of the impacts of COVID-19 on the top-20 metropolitan cities of Asia-Pacific
Source: Sci Rep. 2021 Oct 13;11:20339. doi: 10.1038/s41598-021-99546-9 (PMC8514535; doi:10.1038/s41598-021-99546-9)
Supplement: Supplementary file 2 — Supplementary Information 2. [file 41598_2021_99546_MOESM2_ESM.pdf]

Supplementary Material for

Environmental Spatial Heterogeneity of the Impacts of COVID-19 on the Top-20 Metropolitan Cities of Asia-Pacific

Ghaffar Ali[1], Sawaid Abbas[2,*], Faisal Mueen Qamer[3], Syed Muhammad Irteza[4]

[1] College of Management, Shenzhen University, Shenzhen, 518060, Guangdong, China

[2] Department of Land Surveying and Geoinformatics, The Hong Kong Polytechnic University, Hong Kong

[3] International Center for Integrated Mountain Development (ICIMOD), Kathmandu 44700, Nepal

[4] Remote Sensing, GIS and Climatic Research Lab (RSGCRL), National Center of GIS and Space Applications, University of the Punjab, Lahore, Pakistan

*Correspondence: sawaid.abbas@gmail.com (S.A)

ORCID (Corresponding Author): https://orcid.org/0000-0003-3417-217X

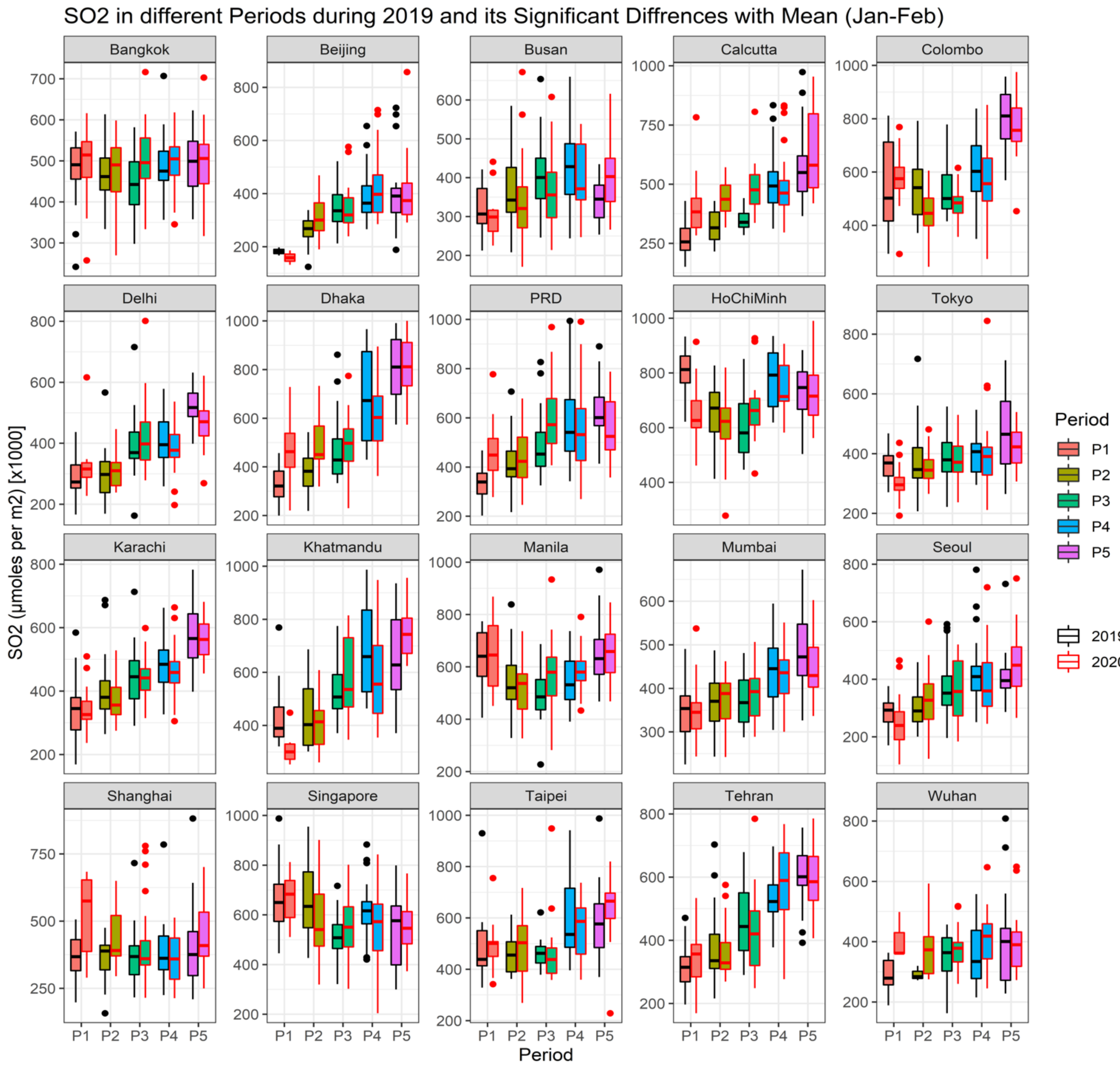

Fig. S1. Boxplot indicating changes in SO2 concentration of the cities during the five different periods in 2019 and 2020.

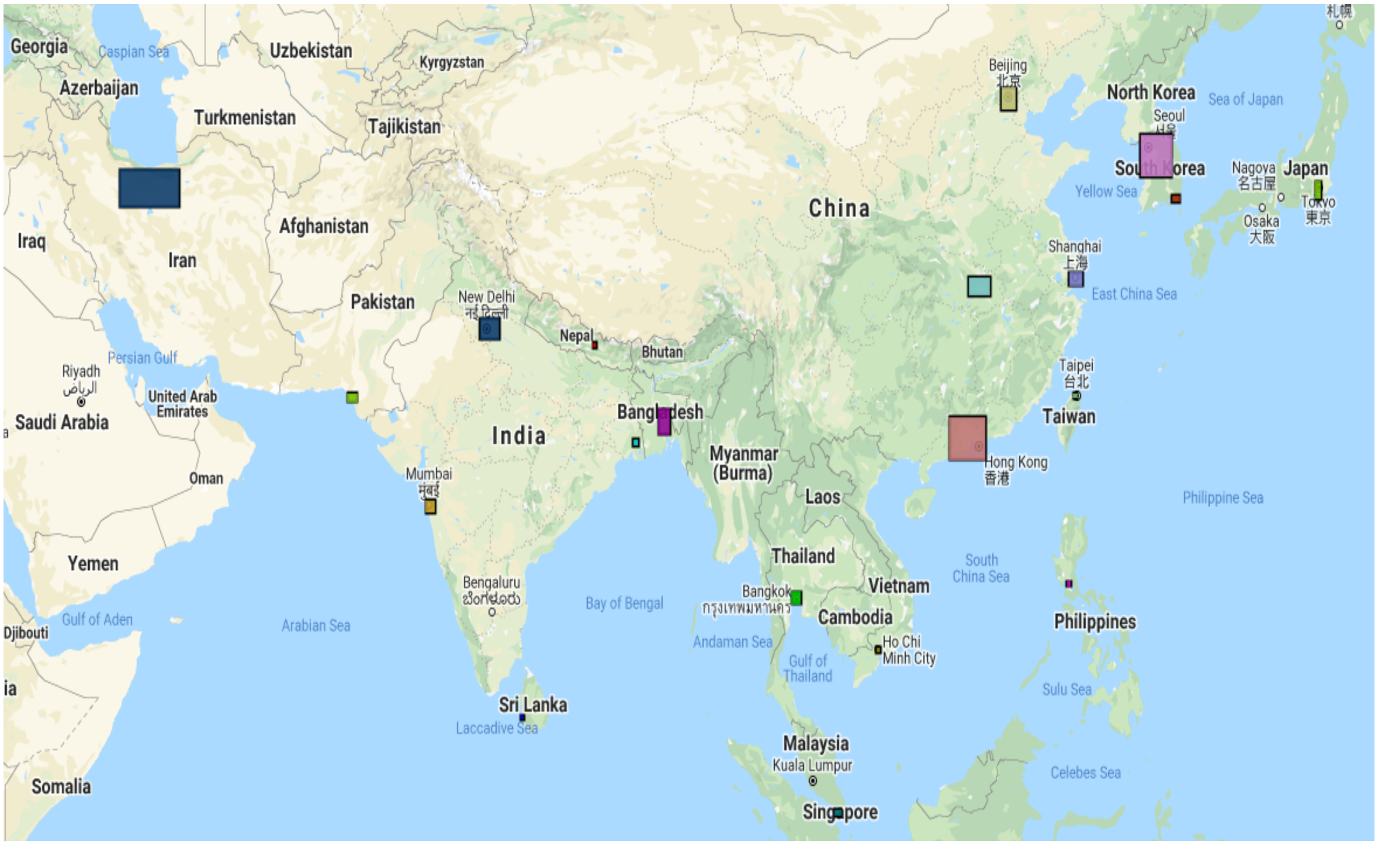

Fig. S2. Location and distribution of the study sites (the Top-20 metropolitan cities in the Asia Pacific region). Maps were created through Google Earth Engine (https://earthengine.google.com/ V. 2020) by a co-author, Sawaid Abbas.
